# Exercise-Induced Pulmonary Hypertension: What Is New?

**DOI:** 10.3390/jcdd13080366

**Published:** 2026-08-03

**Authors:** Ronald Zolty

**Affiliations:** Cardiology, University of Nebraska Medical Center, Omaha, NE 68198, USA; ronald.zolty@unmc.edu

**Keywords:** exercise, pulmonary hypertension, new definition

## Abstract

Exercise-induced pulmonary hypertension (EIPH) represents an abnormal pulmonary vascular response to increased blood flow during exercise, best characterized by a disproportionate rise in pulmonary arterial pressure relative to cardiac output. The shift from fixed pressure thresholds to flow-adjusted metrics, particularly the mPAP–cardiac output slope, reflects a more physiologically sound approach and is now incorporated in the 2022 guidelines of the European Society of Cardiology/European Respiratory Society as well as the World Symposium on PH. EIPH is a hemodynamic phenotype, not a standalone disease. Accumulating evidence demonstrates that EIPH has important diagnostic and prognostic implications. It is associated with reduced exercise capacity, increased risk of adverse cardiovascular outcomes, and reduced survival, even in patients with normal resting hemodynamics. Exercise hemodynamic abnormalities may also identify early or latent pulmonary vascular disease and early left heart dysfunction, particularly in at-risk populations such as systemic sclerosis, HFpEF, and genetic predisposition. In this review, we provide the current definition of EIPH as well as an overview of the prognostic implications and management of exercise-induced PH in cardiac and pulmonary diseases.

## 1. Introduction

Exercise-induced pulmonary hypertension (EIPH) is a pathological hemodynamic condition defined by abnormally elevated pulmonary artery pressures or pulmonary vascular resistance during exercise, despite normal resting values [1]. EIPH is not a formal standalone diagnosis in current guidelines, but rather a physiologic abnormality that can indicate early pulmonary vascular disease or left-heart disease before resting pulmonary hypertension appears. As stated by the 2022 European Society of Cardiology/European Respiratory Society guidelines, EIPH is defined by a mean PA (mPAP)/cardiac output slope > 3 mmHg·L^−1^·min^−1^ from rest to peak exercise [2,3].

The pulmonary circulation is normally a highly compliant, low-resistance system capable of accommodating large increases in blood flow during physical activity through vascular recruitment and distension. Consequently, in healthy individuals, pulmonary artery pressures increase only modestly during exercise despite a substantial increase in cardiac output. When this adaptive response is impaired, pulmonary pressures may rise disproportionately with exercise, suggesting early pulmonary vascular dysfunction or cardiac dysfunction [2,4,5,6,7].

Exercise stress testing of pulmonary circulation has emerged as a valuable approach for identifying early or latent pulmonary vascular or cardiac disease that may not be detectable under resting conditions.

Exercise hemodynamic testing can be performed using several approaches. Invasive cardiopulmonary exercise testing with right heart catheterization (RHC) remains the reference method, allowing direct measurement of pulmonary artery pressures (PAPs), pulmonary artery wedge pressure (PAWP), cardiac output (CO), and derived parameters such as pulmonary vascular resistance (PVR) during graded exercise. These measurements permit differentiation between abnormalities arising from the pulmonary vasculature and those resulting from elevated left-sided filling pressures, which are commonly observed in conditions such as heart failure with preserved ejection fraction (HFpEF). Non-invasive modalities, particularly exercise echocardiography, may serve as useful screening tools but provide less precise hemodynamic information. Doppler-derived estimates of pulmonary artery systolic pressure during exercise may raise suspicion of abnormal pulmonary hemodynamics, although confirmation typically requires invasive assessment.

This review summarizes the current evidence on EIPH, with a focus on its evolving definition, physiological basis, prognostic implications, and potential management strategies. Particular emphasis is placed on the historical development of the EIPH concept and the rationale underlying its reintroduction in the 2022 ESC/ERS pulmonary hypertension guidelines [2]. We further discuss the diagnostic and prognostic value of exercise hemodynamics and provide a disease-specific perspective on EIPH in pulmonary arterial hypertension, systemic sclerosis, HFpEF, chronic lung disease, and chronic thromboembolic pulmonary hypertension (CTEPH).

## 2. Physiological and Pathophysiological Response of the Pulmonary Circulation to Exercise

In healthy individuals, exercise induces a substantial increase in cardiac output, which may rise three- to five-fold above resting levels. Pulmonary circulation normally accommodates this increase in flow through two principal mechanisms: recruitment of previously under-perfused pulmonary capillaries and distension of already perfused vessels. These adaptive processes result in a marked increase in pulmonary vascular capacitance and compliance, allowing the pulmonary vascular bed to accommodate large increases in flow with only modest increases in pulmonary artery pressures [5,8,9,10].

Therefore, pulmonary vascular resistance typically decreases or remains stable during exercise, and the rise in mean pulmonary artery pressure (mPAP) is relatively limited compared with the increase in cardiac output. This efficient coupling between pressure and flow is a defining feature of normal pulmonary vascular physiology.

The pathophysiology underlying abnormal pulmonary hemodynamic responses during exercise may involve several mechanisms. In early pulmonary vascular disease, structural and functional alterations in the pulmonary arteries—including endothelial dysfunction, impaired vasodilation, and reduced vascular compliance—limit the capacity of the pulmonary circulation to accommodate increased flow. This results in a disproportionate rise in pulmonary artery pressures relative to cardiac output. In other cases, abnormal exercise hemodynamics may reflect occult left heart disease, where elevated left ventricular filling pressures are transmitted backward into the pulmonary circulation during exercise [2,4,6,7,11,12,13,14].

## 3. Historical Definition of Exercise-Induced Pulmonary Hypertension

Pulmonary hemodynamics during exercise has been a subject of interest for several decades. The relevance of exercise hemodynamics was formally addressed at the 1973 World Health Organization (WHO) meeting on primary pulmonary hypertension (PH), where early observations suggested that pulmonary arterial pressures remain relatively stable with only modest elevation during exercise in healthy individuals. Based on these data and expert opinion, it was postulated that mPAP should not exceed 30 mmHg during exercise, forming the basis for the initial concept of exercise-induced pulmonary hypertension [15].

Building on this early framework, a hemodynamic definition of EIPH was later incorporated into the 2004 guidelines of the European Society of Cardiology (ESC), where it was defined as mPAP > 30 mmHg during exercise in the presence of normal resting pulmonary pressures [16]. This definition represented one of the first formal attempts to standardize the identification of abnormal pulmonary vascular responses to exercise in clinical practice. However, this definition was critically reassessed at the 4th World Symposium on Pulmonary Hypertension in Dana Point (2008), [17] where accumulating evidence from systematic analyses demonstrated that exercise pulmonary hemodynamics are strongly influenced by several factors, including age, cardiac output, level of exertion, and left ventricular filling pressures. Importantly, healthy older individuals may exceed the 30 mmHg threshold during intense exercise without evidence of pulmonary vascular disease, thereby limiting the specificity of a fixed pressure threshold [18]. Consequently, this definition was considered inadequate, and exercise-induced PH was removed from the formal hemodynamic classification of PH in subsequent guidelines [17,19]. At the same time, there was recognition of the need for further research focusing on pressure–flow relationships, which has since generated the development of contemporary, flow-adjusted definitions of EIPH.

In 2022, after decades of accumulating evidence, the ESC/ERS pulmonary hypertension guidelines reintroduced the concept of exercise PH, defining it as an mPAP–cardiac output (CO) slope > 3 mmHg·L^−1^·min^−1^ from rest to peak exercise. This flow-adjusted definition accounts for the physiological increase in pulmonary pressures with rising cardiac output and provides a more accurate framework for identifying abnormal pulmonary vascular response during exercise [2,3].

## 4. Contemporary Concept: Pressure–Flow Relationships and New Definition

Contemporary investigations have emphasized the importance of evaluating pulmonary pressures in relation to cardiac output rather than relying solely on absolute pressure values. The pulmonary circulation is fundamentally a low-resistance, high-compliance vascular system, and interpretation of exercise hemodynamics therefore requires assessment of the dynamic interaction between pressure and flow.

Recent EIPH research has increasingly focused on the mPAP–cardiac output slope, which reflects the increase in pulmonary artery pressure per unit increase in cardiac output during exercise. In healthy individuals, this slope is typically ≤3 mmHg per L/min, indicating an efficient pulmonary vascular response to increased flow. A steeper slope suggests an abnormal pulmonary vascular response and may indicate early pulmonary vascular disease.

The mPAP/CO slope is age-dependent, with an upper limit of normal ranging from 1.6 to 3.3 mmHg/L/min in the supine position, and a slope > 3 mmHg/L/min is not physiological in subjects aged <60 years [2].

**Figure 1:**
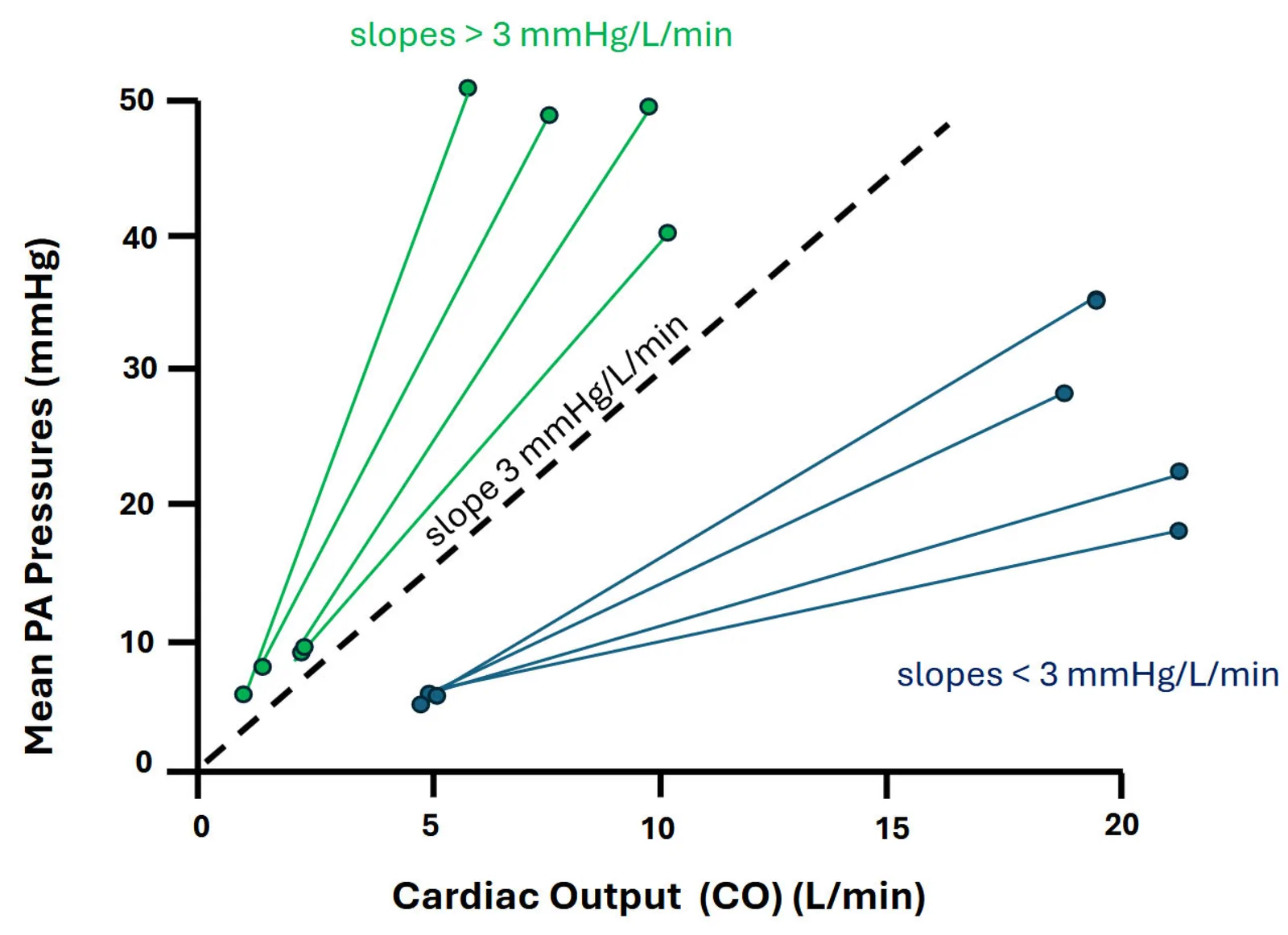
During exercise, cardiac output increases substantially. In healthy individuals, the pulmonary circulation accommodates this increased flow through vascular recruitment and distension, resulting in only a modest rise in pulmonary artery pressure. Consequently, the mPAP/CO slope remains relatively low (<3 mmHg/L/min). Patients with pulmonary vascular disease, reduced pulmonary vascular compliance, or abnormal left-heart filling pressures exhibit a disproportionate increase in pulmonary artery pressure relative to flow, resulting in an elevated mPAP/CO slope (>3 mmHg/L/min).

The 7th WSPH in 2026 also endorsed the exercise-induced PH definition already established by the 2022 ESC/ERS Guidelines: exercise PH is defined as mPAP/cardiac output (CO) slope > 3 mmHg/L/min between rest and exercise in patients with normal mPAP at rest [2,20] (Figure 1).

## 5. Differentiating Pre-Capillary from Post-Capillary Exercise PH

The critical distinction in assessing EIPH is whether the exercise PH is pre-capillary (pulmonary vascular disease) or post-capillary (left heart disease). The pulmonary artery wedge pressure (PAWP)/cardiac output (CO) slope describes how much left-sided filling pressure rises for a given increase in cardiac output during exercise. It is calculated as PAWP/CO slope = (PAWPpeak − PAWPrest)/(COpeak − COrest).

**Figure 2:**
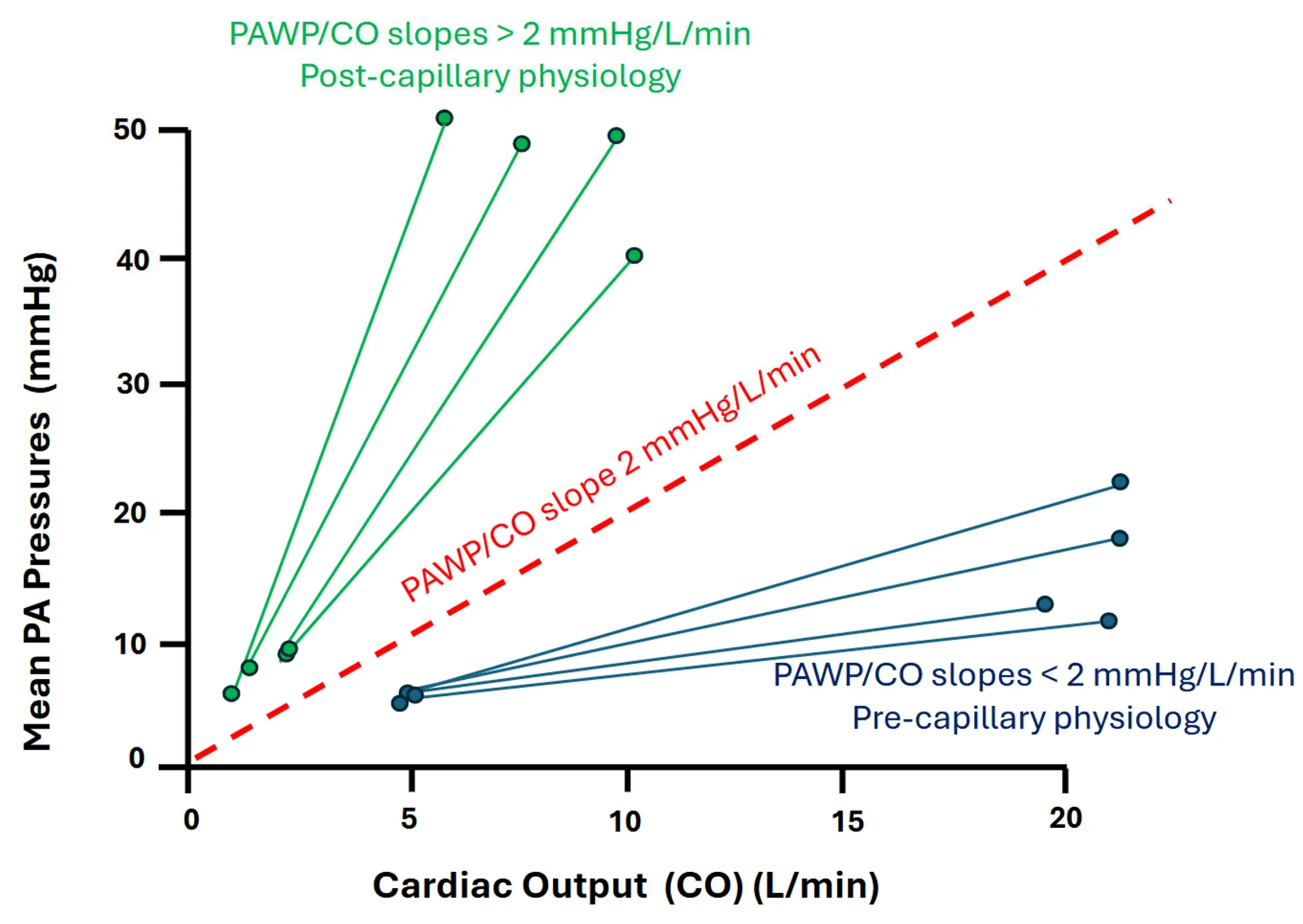
During exercise, cardiac output increases substantially to meet metabolic demands. In healthy individuals, the left ventricle and left atrium accommodate this increased blood flow with only a modest rise in filling pressures. Consequently, PAWP increases only slightly relative to the increase in cardiac output. When the left ventricle is stiff or has impaired diastolic reserve, as occurs in heart failure with preserved ejection fraction (HFpEF), filling pressures rise disproportionately.

The PAWP/CO slope is the key differentiating parameter [2,21,22]. A PAWP/CO slope < 2 mmHg/L/min suggests a pre-capillary phenotype, reflecting preserved left-sided filling pressures during exercise and pointing toward pulmonary vascular dysfunction, while a PAWP/CO slope > 2 mmHg/L/min is consistent with abnormal left heart filling pressures (e.g., HFpEF) and upstream transmission of pressure to the pulmonary circulation. (Figure 2).

**Figure 3:**
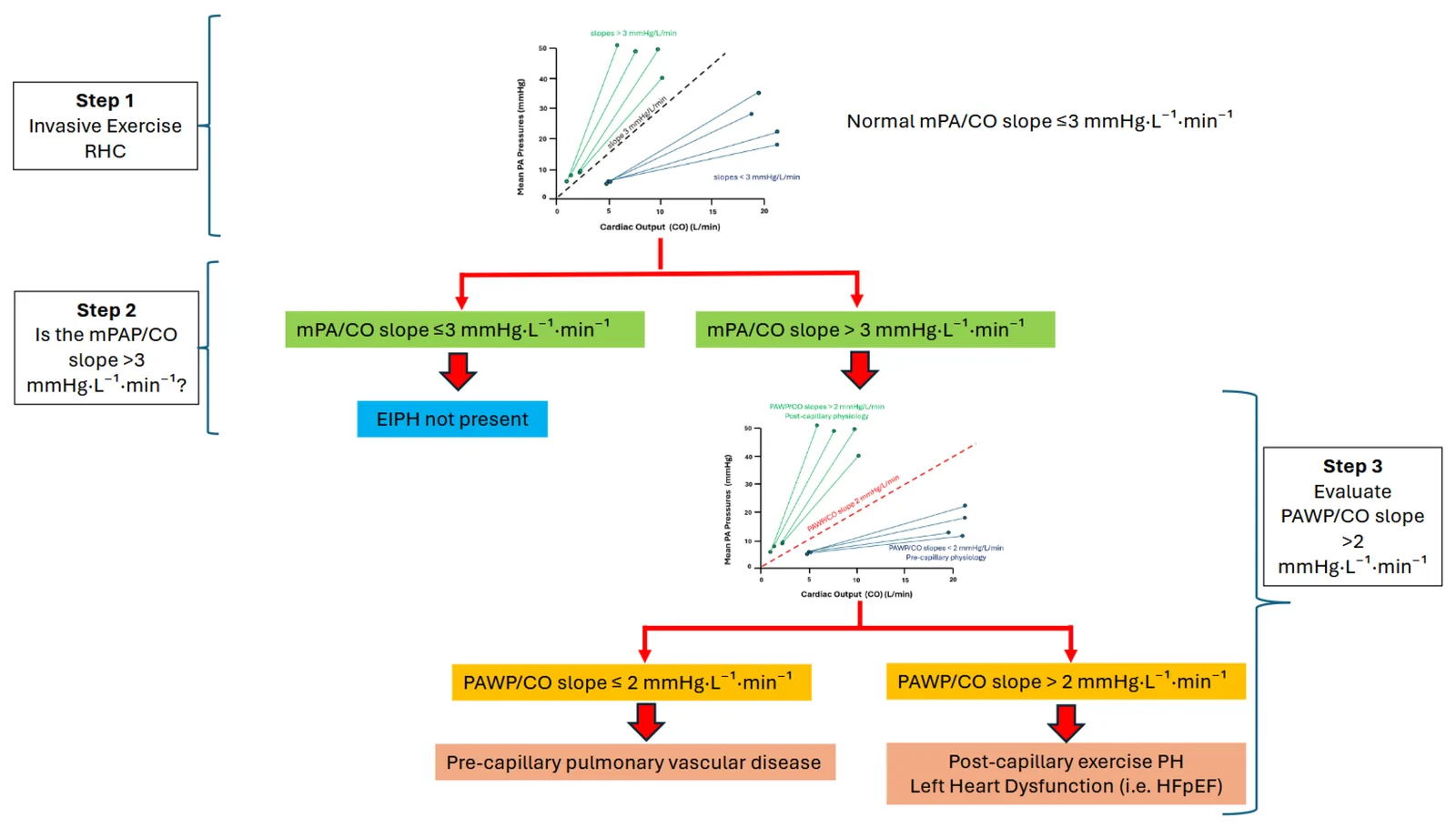
Algorithm for interpreting exercise hemodynamics. The algorithm is structured around the mPAP/CO slope and PAWP/CO slope, consistent with the 2022 ESC/ERS guidelines. **Step 1**: Perform exercise RHC. **Step 2**: Is the mPAP/CO slope > 3 mmHg·L^−1^·min^−1^? If No (≤3 mmHg·L^−1^·min^−1^): normal pulmonary vascular response to exercise and EIPH not present. If Yes (>3 mmHg·L^−1^·min^−1^): Exercise PH is present; proceed to assessment of PAWP/CO slope. **Step 3**: Evaluate PAWP/CO slope: If PAWP/CO slope ≤ 2 mmHg·L^−1^·min^−1^ it suggests a pre-capillary pattern such as early pulmonary vascular disease. If PAWP/CO slope > 2 mmHg·L^−1^·min^−1^ it suggests a post-capillary pattern such as HFpEF, left atrial dysfunction, or valvular heart disease.

A practical algorithm for interpreting exercise hemodynamics and differentiating pre-capillary from post-capillary exercise pulmonary hypertension (PH) can be structured around the mean pulmonary artery pressure–cardiac output (mPAP/CO) slope and pulmonary artery wedge pressure–cardiac output (PAWP/CO) slope, consistent with the 2022 ESC/ERS guidelines [2]. An mPAP/CO slope > 3 mmHg·L^−1^·min^−1^ from rest to peak exercise establishes the presence of exercise PH. Once exercise PH has been identified, assessment of the PAWP/CO slope helps determine the underlying mechanism. A PAWP/CO slope > 2 mmHg·L^−1^·min^−1^ suggests an abnormal rise in left-sided filling pressures and is indicative of a post-capillary phenotype, most commonly associated with HFpEF, diastolic dysfunction, or other forms of left heart disease. In contrast, an mPAP/CO slope > 3 mmHg·L^−1^·min^−1^ in the presence of a PAWP/CO slope ≤ 2 mmHg·L^−1^·min^−1^ suggests a pre-capillary phenotype, reflecting early pulmonary vascular disease. This pressure–flow approach provides a physiologically grounded framework for identifying the dominant mechanism underlying exercise intolerance and abnormal pulmonary hemodynamics (Table 1, Figure 3).

Additionally, a PAWP > 25 mmHg during supine exercise has been recommended for diagnosing HFpEF [2]. Left heart disease can also be ascertained by a PAWP above 20 mmHg at a cardiac output < 10 L/min during exercise [7].

## 6. Pulmonary Hemodynamics During Exercise in Healthy Subjects

The ranges of normal pulmonary hemodynamics during exercise have been extensively investigated in recent years through meta-analyses and comprehensive systematic reviews, providing detailed insights into how pulmonary artery pressures respond to increased cardiac output across different populations.

In one such analysis, hemodynamic data from 1187 healthy subjects across 47 studies were compiled and stratified according to sex, age, ethnicity, geographic origin, body position, and exercise intensity. The study confirmed that mPAP during exercise is strongly influenced by age and the level of exertion, highlighting the limitations of using fixed pressure thresholds to define abnormal responses [18].

At mild exercise levels, participants aged ≥50 years exhibited significantly higher mPAP values than those aged <50 years (29 ± 8 mmHg versus 19 ± 5 mmHg, *p* < 0.001). At maximal exercise, older individuals frequently exceeded an mPAP of 30 mmHg, whereas approximately 20% of younger subjects surpassed this threshold as well. Importantly, in these younger participants, elevated mPAP values were associated with very high cardiac output, reflecting physiological adaptations rather than pathological changes.

A second meta-analysis by Zeder et al. included 250 healthy subjects from 11 studies. Results demonstrated that the mPAP/CO slope increased progressively with age. Specifically, mean slopes were: 0.8 ± 0.4 Wood units (WU) in individuals around 30 years of age (ULN 1.6 WU), 1.6 ± 0.2 WU in individuals around 50 years of age (ULN 2.1 WU), and 2.4 ± 0.5 WU in individuals around 70 years of age (ULN 3.3 WU) [21].

These findings indicate that an mPAP/CO slope exceeding 3 WU is generally considered pathological, even in elderly individuals, as it exceeds the physiological upper limit of normal for age.

Similarly, the pulmonary artery wedge pressure (PAWP)/CO slope increased with age as well, ranging from 0.3 ± 0.2 WU (ULN 0.6 WU) in younger adults (~30 years) to 1.4 ± 0.2 WU (ULN 1.8 WU) in older adults (~70 years) [21]. This illustrates that age-adjusted evaluation of pressure–flow relationships is essential to distinguish normal physiological responses from early or latent pulmonary vascular disease.

## 7. Prognosis and Clinical Significance of EIPH

EIPH has important prognostic implications even when resting pulmonary pressures are normal. In the landmark PEX-NET study, which included 764 patients with normal or mildly elevated resting mPAP, an mPAP/CO slope > 3 mmHg·L^−1^·min^−1^ during exercise was independently associated with increased mortality. After adjustment for age, sex, and resting hemodynamics, this abnormal pressure–flow response remained a significant predictor of death (hazard ratio 2.04; 95% CI 1.16–3.58). These findings highlight the prognostic value of exercise hemodynamic assessment in identifying patients with early pulmonary vascular or cardiopulmonary dysfunction that may not be evident from resting measurements alone [22].

In this large study, a steeper mPAP/CO slope was strongly associated with increased mortality and adverse clinical events, independent of resting pulmonary pressures, age, and underlying comorbidities. Similarly, lower peak cardiac index during exercise correlated with poor prognosis, reflecting limited cardiovascular reserve and impaired ability to augment cardiac output in response to stress.

In another large cohort study, Ho et al. [23] evaluated 714 subjects to examine the prognostic significance of EIPH, defined as resting mPAP ≤ 20 mmHg with an mPAP/CO slope > 3 mmHg·L^−1^·min^−1^ during exercise. The primary endpoint was a composite of all-cause mortality or cardiovascular hospitalization. The presence of exercise PH was associated with a two-fold increased risk of events after adjusting for clinical confounders, including gender, age, history of heart failure, hypertension, interstitial lung disease (ILD), COPD, and smoking status. Prognostically, subjects with resting PH at rest had the poorest survival, followed by those with EIPH, while individuals without resting or EIPH had the best outcomes.

Beyond the mPAP/CO slope, the study demonstrated that trans-pulmonary gradient (TPG)/CO and PAWP/CO slopes were also independently predictive of survival, reinforcing the value of flow-adjusted hemodynamic metrics in assessing pulmonary vascular function and risk stratification [23].

## 8. Prognosis and Clinical Significance of PAWP/CO Slope > 2

A study by Eisman et al. evaluated exercise pulmonary hemodynamics in 110 subjects presenting with dyspnea and suspected HFpEF, who had normal resting PAWP and left ventricular ejection fraction. A PAWP/CO slope > 2 WU was strongly associated with adverse clinical outcomes, including cardiovascular death, heart failure hospitalization, or progression to abnormal resting PAWP on future RHC. These findings highlight the prognostic value of flow-adjusted PAWP responses during exercise in detecting early post-capillary pulmonary vascular impairment and identifying patients at risk for progression to overt HFpEF [24].

## 9. Knowledge Gaps

The natural history of exercise-induced PH and its progression to manifest disease remains an important area of investigation. As noted in the literature, serial rest and exercise hemodynamic measurements are needed to further characterize the natural history of EIPH, including its relationship to long-term PH/PAH progression [23].

## 10. Upright Versus Supine Bike Exercise and EIPH

Body position significantly affects pulmonary hemodynamics during exercise, with upright exercise producing approximately 10% higher PVR and higher mean pulmonary artery pressures compared to supine exercise, even after adjusting for workload differences. Both positions have distinct advantages and limitations that should be considered when interpreting exercise testing for PH [25].

Importantly, upright exercise allowed patients to achieve higher workloads (53 ± 26 vs. 33 ± 22 watts), providing a broader physiological window for assessment [25]. Upright exercise combined with metabolic cart measurements provides the most complete cardiopulmonary evaluation, particularly when assessing peak exercise capacity and identifying contributors to exercise limitation beyond hemodynamics [26].

The mPAP/CO slope > 3 mmHg/L/min threshold for defining EIPH was derived primarily from supine exercise data [26]. The 2022 ESC/ERS Guidelines note that the upper limit of normal for mPAP/CO slope ranges from 1.6 to 3.3 mmHg/L/min in the supine position [3]. Given that upright exercise produces higher mPAP/CO slopes, body position must be considered and reported when assessing for EIPH [25].

According to the 2026 American Heart Association Scientific Statement on standardization of invasive hemodynamic protocols, an mPAP–cardiac output (CO) slope > 3 mmHg·L^−1^·min^−1^ is an appropriate threshold for the diagnosis and prognostic assessment of exercise pulmonary hypertension, irrespective of body position [26].

A study in 26 healthy volunteers found that body position did not affect the slope of mPAP–cardiac output relationships (average 1.5 ± 0.4 mmHg/L/min across all positions), maximum oxygen uptake, or total pulmonary vascular resistance when results were expressed as mPAP-CO relationships [27]. This suggests that pressure-flow relationships may be position-independent, though absolute pressures differ.

## 11. Populations at Risk for EIPH

EIPH may precede the development of overt resting PH and is associated with reduced survival and diminished exercise capacity [7]. In patients with unexplained chronic dyspnea, EIPH predicts worse cardiovascular event-free survival, even in the absence of resting PH or established cardiovascular disease [23]. EIPH has been described in several conditions, including left heart disease, systemic sclerosis, BMPR2 mutation carriers, and chronic lung disease, including COPD, interstitial lung disease, and chronic thromboembolic disease [7].

### 11.1. Cardiovascular Disease and EIPH

Patients with left heart conditions, particularly those with HFpEF, frequently demonstrate EIPH. In a large cohort of 714 patients with chronic dyspnea, those with EIPH (41% of the cohort) had significantly higher rates of diabetes (22% vs. 12%), prior heart failure symptoms (20% vs. 7%), and elevated NT-proBNP levels (median 154 vs. 52 pg/mL) [23]. Patients with HFpEF and normal resting PAWP typically show steep increases in both mPAP and PAWP during exercise, with a PAWP/CO slope > 2 mmHg/L/min [2].

#### HFpEF and EIPH

EIPH is a critical diagnostic feature in HFpEF, even in patients who have normal resting filling pressures but develop elevated pulmonary capillary wedge pressure (PCWP) only during exertion [28]. Exercise hemodynamics are essential for both diagnosing HFpEF and distinguishing isolated postcapillary PH (IpcPH) from combined pre- and postcapillary PH (CpcPH).

Pulmonary hypertension is present in approximately 80% of HFpEF patients and is associated with increased mortality [29].

The 2022 ESC/ERS Guidelines and the American Heart Association/JACC Scientific Statement define key hemodynamic criteria for abnormal exercise responses in left ventricular (LV) dysfunction [2,28]:

- PAWP ≥25 mmHg during supine exercise supports a diagnosis of HFpEF;
- PAWP/CO slope > 2 mmHg·L^−1^·min^−1^ indicates an abnormal rise in left-sided filling pressures;
- mPAP/CO slope > 3 mmHg·L^−1^·min^−1^ combined with PAWP/CO slope > 2 mmHg·L^−1^·min^−1^ is consistent with post-capillary exercise PH.

These criteria emphasize a pressure–flow–based approach, allowing differentiation between pulmonary vascular and left heart–mediated mechanisms of exercise intolerance.

RHC with exercise is considered the gold standard test for HFpEF particularly in patients with intermediate H2FPEF scores (2–5) [30].

The key hemodynamic distinction between precapillary versus postcapillary PH during exercise lies in the PAWP/CO slope [29]. In precapillary PH (early pulmonary vascular disease), the pulmonary pressures rise disproportionately while left-sided filling pressures remain relatively stable; the mPAP/CO slope is >3 mmHg/L/min with PAWP/CO slope < 2 mmHg/L/min. Alternatively, in postcapillary PH (i.e., HFpEF), both mPAP and PAWP rise steeply during exercise, with a PAWP/CO slope > 2 mmHg/L/min because elevated left ventricular filling pressures are transmitted backward into the pulmonary circulation.

A distinct pathophysiological phenotype has been identified in HFpEF patients with disproportionate exercise-induced PH (mPAP/CO slope > 5.2 mmHg/L/min) [31]. Compared to HFpEF patients without DEi-PH, the individuals with DEIPH demonstrate a worse exercise capacity (lower peak VO_2_), a depressed RV systolic function, and impaired RV–pulmonary artery coupling, greater right-sided congestion and worse ventilatory efficiency (higher VE/VCO_2_), and finally, higher rates of mortality and HF events (log-rank *p* = 0.0002) [31]. Importantly, these abnormalities are not apparent at rest, with similar resting RV systolic function and PH severity between groups, emphasizing the critical role of exercise testing [31].

Exaggerated exercise PH in HFpEF as seen in DEIPH contributes to ventilation-perfusion mismatch, causing lower arterial oxygen tension (PaO_2_) at peak exercise, higher dead space to tidal volume ratio, increased VE/VCO_2_ slope (ventilatory inefficiency), and greater perceived breathlessness at equivalent workloads [32].

CpcPH, defined by PVR ≥2–3 WU in the setting of elevated PAWP, may be more frequent in HFpEF than HFrEF [33]. Patients with CpcPH-HFpEF demonstrate unique exercise limitations with a greater increase in right atrial pressure during exercise, an inability to augment cardiac output appropriately, and marked limitation in aerobic capacity [34].

Exercise hemodynamic testing enables identification of HFpEF patients with latent pulmonary vascular disease—those with normal resting PVR but abnormal exercise PVR response—who represent a higher-risk phenotype [35]. This phenotyping is increasingly important as therapies targeting the pulmonary vasculature may have differential effects in IpcPH versus CpcPH [36].

### 11.2. Systemic Sclerosis and EIPH

Systemic sclerosis (SSc) represents the highest-risk connective tissue disease population for developing PAH. In a recent study of SSc patients with exercise intolerance, 44% exhibited exercise PH, with many showing evidence of latent HFpEF [37]. The prevalence of resting PAH in SSc cohorts ranges from 5 to 19%, with an annual incidence of developing PAH of 0.7–1.5% [2]. EIPH in SSc patients portends future development of manifest PH and is associated with worse clinical outcomes [23].

In a cohort of 80 patients with resting mPAP < 25 mmHg, invasive exercise RHC was performed, and patients were followed for over 10 years. The study found that exercise-derived pulmonary vascular measures, including pulmonary vascular resistance (PVR), mPAP/CO slope, and TPG/CO slope, were significant predictors of 10-year mortality, whereas resting hemodynamic variables had no prognostic value. Notably, an mPAP/CO slope of 3.5 WU was identified as the optimal threshold for classifying long-term mortality. Importantly, none of the patients with an mPAP/CO slope below 3.5 WU died or developed PH during follow-up, emphasizing the clinical relevance of flow-adjusted exercise hemodynamic assessment for early risk stratification in systemic sclerosis [38].

### 11.3. Genetic Predisposition and BMPR2 Mutations and EIPH

Carriers of BMPR2 gene mutations who do not yet have clinically overt PH are at increased risk of developing EIPH. Exercise-induced PH in this population may represent an early manifestation of pulmonary vascular dysfunction and can precede the onset of overt PAH, highlighting the potential value of exercise hemodynamic testing for early detection and risk stratification in genetically predisposed individuals [7,23].

EIPH is prevalent in asymptomatic BMPR2 mutation carriers, with the DELPHI-2 study demonstrating that 21.8% (12/55) of carriers had EIPH at baseline despite normal resting hemodynamics and echocardiography [38]. This finding supports the concept that abnormal pulmonary vascular responses to exercise may represent early, subclinical disease expression in this high-risk population.

BMPR2 mutations are the most common genetic cause of heritable PAH, accounting for approximately 80% of familial cases and up to 20% of sporadic PAH [39]. Carriers have a lifetime risk of developing overt PAH of approximately 20%, with higher penetrance in females (42%) compared to males (14%) [3]. The incomplete penetrance suggests that a “second hit”—either genetic or environmental—may be necessary to trigger disease manifestation [40].

Some asymptomatic BMPR2 mutation carriers have been shown to have abnormal increases in PAPs with exercise, suggesting subclinical pulmonary vascular dysfunction even before overt disease develops [41]. However, some invasive studies have yielded mixed results. In one study using gold-standard invasive hemodynamics, only 1 of 8 BMPR2 carriers had an abnormal mPAP/CO slope (>3 mmHg/L/min) during invasive exercise, similar to controls [42]. Nevertheless, in the same study, subjects submitted to hypoxia with a mixture of 12% oxygen and 88% nitrogen, 3 of 8 carriers (37.5%) developed abnormal mPAP/CO slopes compared to none of the controls, suggesting that hypoxia may unmask latent pulmonary vascular dysfunction [42].

Phenotyping studies have revealed that even unaffected BMPR2 carriers demonstrate subclinical cardiac abnormalities with smaller biventricular volumes (lower RV and LV end-diastolic volumes), higher pulmonary arterial afterload (arterial elastance 0.27 vs. 0.15 mmHg/mL, *p* < 0.001), and impaired RV–pulmonary artery coupling (Ees/Ea ratio 1.36 vs. 2.24, *p* = 0.006) [43]. These findings were validated in transgenic Bmpr2^Δ71Ex1/+^ rat models, suggesting an intrinsic cardiac phenotype associated with BMPR2 haploinsufficiency [43].

Evidence suggests that EIPH may predict progression to overt PAH in BMPR2 carriers. In a large German family with a BMPR2 mutation followed for a mean of 12 years, 19 family members had normal resting pulmonary pressures with evidence of EIPH [44]. During follow-up, only those members who carried both the mutation and had pulmonary hypertensive exercise responses developed manifest PAH, suggesting exercise PH may be an additional risk factor for disease penetrance [44].

### 11.4. Pulmonary Disease and EIPH

Chronic lung diseases, including COPD (18% vs. 5%) and interstitial lung disease (13% vs. 6%), are significantly more prevalent in patients with exercise PH compared to those with normal exercise hemodynamics [24]. Patients with chronic thromboembolic disease are also at elevated risk [7].

#### 11.4.1. COPD and EIPH

EIPH is highly prevalent in COPD patients, even among those with normal resting pulmonary pressures. COPD patients demonstrate significantly steeper mPAP/cardiac output (CO) slopes during exercise compared to controls, reflecting impaired pulmonary vascular reserve and loss of vascular distensibility [45,46,47].

A study by Sassmann et al. found that COPD patients without significant resting PH (mPAP < 25 mmHg) had a median mPAP/CO slope of 6.9 mmHg/L/min compared to 3.7 mmHg/L/min in matched controls (*p* = 0.007) [45]. During exercise, COPD patients reached significantly higher mPAP (47 vs. 38 mmHg) and PVR (3.1 vs. 1.7 WU) despite achieving lower peak workloads [45].

Hilde et al. examined 98 COPD outpatients with exercise invasive RHC and found that 45% met criteria for exercise-induced PH (mPAP/CO slope > 3 mmHg/L/min) [46,48]. Importantly, patients without resting PH showed similar abnormal hemodynamic responses to exercise as those with established PH, including increased PVR, reduced pulmonary artery compliance, and steeper mPAP/CO slopes [46].

The pathophysiology of EIPH in COPD involves multiple factors [49,50], including (a) loss of vascular distensibility and recruitment capability—even under moderate exercise, COPD patients show rapid rises in mPAP, indicating impaired ability to accommodate increased flow [47]; (b) pulmonary vascular remodeling including intimal hyperplasia, smooth muscle hypertrophy, and medial thickening causing reduced luminal cross-sectional area and increased PVR [49]; (c) endothelial dysfunction which is present in all stages of COPD and even in smokers with normal lung function, leading to imbalance of vasoconstrictive and vasodilating factors [49]; (d) chronic pulmonary vascular inflammation with CD8+ T-cell infiltration and inflammatory mediators contributing to vascular remodeling [49]; and (e) dynamic hyperinflation. The increased intrathoracic pressure during exercise contributes to mPAP elevation, recognizable by concomitant increases in right atrial pressure [2,51].

The mPAP/CO slope negatively correlates with exercise capacity, including peak oxygen uptake (r = −0.46, *p* = 0.007) and 6 min walk distance (r = −0.46, *p* = 0.001) [45]. COPD patients with EIPH experience a higher “cost” of exercise, with greater increases in oxygen uptake, ventilation, respiratory frequency, heart rate, and lactate for a given workload compared to those with normal hemodynamic responses [48].

Even mild elevations in pulmonary artery pressures are associated with worse outcomes in COPD, including increased risk of acute exacerbation and mortality [49]. A 5-year survival rate of only 36% was reported for COPD patients with mPAP values > 25 mmHg, with pulmonary hemodynamics being a far stronger predictor of survival than FEV_1_ or gas exchange variables [47].

#### 11.4.2. Interstitial Lung Disease (ILD) and EIPH

EIPH is highly prevalent in interstitial lung disease (ILD), with studies demonstrating that pulmonary vascular dysfunction during exercise significantly contributes to exercise limitation even when resting hemodynamics are normal [52].

A recent invasive cardiopulmonary exercise testing study of ILD patients with unexplained exercise intolerance identified several distinct hemodynamic phenotypes demonstrated that 23% had no PH at rest or exercise, 37% had no resting PH but elevated PVR > 2 WU (suggesting subclinical pulmonary vascular disease), 16% had pre-capillary PH, 16% had post-capillary or combined pre- and post-capillary PH, and 9% had EIPH [53].

Exercise testing reveals that pulmonary vascular dysfunction contributes significantly to exercise limitation in ILD, independent of ventilatory impairment [54,55]. ILD patients with EIPH had significantly reduced peak VO_2_ (67 ± 13% predicted) compared to ILD patients without EIPH (81 ± 16% predicted, *p* = 0.016). The mPAP/CO slope ≥3 mmHg/L/min identifies ILD patients with pulmonary vascular dysfunction—in one study, 15 of 27 ILD patients (56%) had abnormal slopes despite similar pulmonary function testing to those without EIPH [55]. Critically, ventilatory reserve was similar between EIPH and ILD versus ILD without EIPH groups at anaerobic threshold (0.32 vs. 0.30, *p* = 0.921) and peak exercise, indicating that the exercise limitation in EIPH and ILD is primarily circulatory rather than ventilatory [54].

Exercise hemodynamics provides important prognostic information in ILD [21,22]. The mPAP/CO slope > 3 WU is independently associated with worse survival (HR 2.04, 95% CI 1.16–3.58, *p* = 0.013), even after adjustment for resting hemodynamics. The peak exercise cardiac output and transpulmonary gradient are independent predictors of prognosis. Finally, across the spectrum from no PH to EIPH to resting pre-capillary PH in ILD, there is a progressive increase in the mPAP/CO slopes (1.9, 3.1, and 5.1, respectively) [51].

#### 11.4.3. EIPH in Chronic Thromboembolic Pulmonary Disease (CTED)/Chronic Thromboembolic Pulmonary Hypertension (CTEPH)

EIPH is highly prevalent in chronic thromboembolic pulmonary disease (CTEPD), occurring in approximately 40–50% of patients with persistent thrombi who have normal or only mildly elevated resting pulmonary pressures [56,57]. Exercise hemodynamics plays a critical role in both the early detection of CTEPD/CTEPH and in assessing residual disease after treatment.

A systematic review and meta-analysis found a pooled prevalence of EIPH of approximately 50% in CTEPD patients without resting PH, with a mean mPAP/CO slope of 4.10 mmHg/L/min [57]. In a prospective cohort of 92 symptomatic patients with chronic thrombi and absent/mild resting PH, 40.2% developed exercise PH (mPAP/CO slope > 3 mmHg/L/min) [56].

EIPH may represent an early marker of pulmonary vascular dysfunction in patients with persistent symptoms after pulmonary embolism. Madonna et al. demonstrated that patients with positive Ventilation/Perfusion (V/Q) scans (indicating persistent thrombi) developed exercise-induced PH at 4 months post-PE, and by 24 months showed progressive RV dysfunction and reduced functional capacity despite maintaining low/intermediate echocardiographic probability of PH at rest [58].

The 2022 ESC/ERS Guidelines recommend exercise RHC to detect early pulmonary vascular disease in patients with unexplained dyspnea and normal resting hemodynamics, noting that exercise hemodynamics provide important prognostic and functional information in patients at risk of CTEPH [2].

Despite significant improvements in resting hemodynamics, abnormal exercise pulmonary vascular responses frequently persist after both pulmonary endarterectomy (PEA) and balloon pulmonary angioplasty (BPA) [59,60].

In a study of 249 CTEPH patients with normalized resting mPAP (<25 mmHg) after BPA, 47% (116/249) had EIPH (mPAP/CO slope > 3.0) [57]. In another long-term follow-up (median 50 months) study, mean mPAP/CO slopes remained elevated at 7.0 ± 5.6 mmHg/L/min after BPA and 4.0 ± 2.3 mmHg/L/min after PEA [60].

The 2022 ESC/ERS Guidelines and 2024 AHA Scientific Statement note that selected symptomatic patients with CTEPD without resting PH can be successfully treated with BPA, with clinical and hemodynamic improvements at rest and exercise [2,61].

## 12. Challenges of Measuring and Interpreting Hemodynamic Parameters During Exercise

The lack of standardized measurement methodology during exercise hemodynamics is a recognized barrier to broader adoption, and the confusion about how to handle respiratory variation is a central part of the problem, particularly in patients with lung disease and obesity. In these patients, there are often wide swings in PAWP and PA pressures, largely related to large swings in intrathoracic pressure.

At rest, the standard is to measure PAWP at end-expiration, when intrathoracic pressure most closely approximates atmospheric pressure [4,26]. However, during exercise, this approach becomes problematic. Respiratory effort increases dramatically, intrathoracic pressure swings widen, and identifying a reliable end-expiratory plateau becomes difficult.

Large swings in intrathoracic pressure during exercise may significantly influence measured pressures, complicating the distinction between true hemodynamic abnormalities and respiratory artifacts. Although correction for pleural pressure using an esophageal balloon may improve accuracy, this approach is invasive, technically demanding, and impractical for routine clinical use. Consequently, uncertainty remains regarding the optimal acquisition, averaging, and interpretation of exercise hemodynamic measurements. These methodological challenges have likely contributed to the limited adoption of exercise right heart catheterization in clinical practice and underscore the need for standardized protocols and reporting methods.

Managing respiratory pressure swings during hemodynamic assessment is one of the most practically challenging aspects of exercise RHC. The 2026 AHA Scientific Statement recommends averaging pressures over ≥3–4 respiratory cycles when respiration varies to manage respiratory swings, and to read tracings manually from high-fidelity paper or screen recordings rather than using automated digital mean values [26]. Also, a JACC review acknowledges that during exercise, averaging PAWP over several respiratory cycles has been widely recommended to better reflect mean left-sided filling pressures [4].

## 13. Management of EIPH

There is currently no approved pharmacologic therapy specifically indicated for EIPH, which is a hemodynamic phenotype and not a standalone disease. Present management of EIPH should focus primarily on supervised exercise-based rehabilitation and treatment of underlying conditions.

Consequently, the routine use of PAH-targeted therapies in EIPH is currently not approved and cannot currently be recommended outside of clinical research settings or specific PH expert centers.

The role of elevated PAWP as the principal determinant of exercise intolerance in HFpEF has recently been questioned. In a study by Sarma et al., acute reduction in exercise PAWP with nitroglycerin significantly lowered left-sided filling pressures but failed to improve exercise capacity [62]. These findings suggest that elevated PAWP during exercise, although an important hemodynamic marker of HFpEF, is not the sole mechanism limiting aerobic performance. Instead, exercise intolerance in HFpEF is likely multifactorial, involving impaired chronotropic reserve, reduced stroke volume augmentation, skeletal muscle dysfunction, endothelial abnormalities, and peripheral oxygen extraction in addition to abnormal left ventricular filling pressures. This study highlights that while exercise PAWP is valuable for diagnosis and phenotyping, reducing filling pressures alone may be insufficient to improve functional capacity. PAWP remains an excellent diagnostic and prognostic marker, but it should not necessarily be viewed as the primary therapeutic target for improving exercise capacity in HFpEF [62].

While specific PAH-targeted therapies such as phosphodiesterase-5 inhibitors, endothelin receptor antagonists, and prostacyclin pathway agents are established therapies for manifest PAH, their role in EIPH remains investigational, and their use for EIPH is presently off-label [63].

A case series of 14 patients with EIPH treated with PH-targeted therapy (endothelin receptor antagonists, phosphodiesterase-5 inhibitors, or both) demonstrated significant improvements in stroke volume augmentation and oxygen consumption at anaerobic threshold on non-invasive cardiopulmonary exercise testing. Specifically, treatment resulted in significant increases in VO2 at anaerobic threshold (0.9 ± 0.3 vs. 0.7 ± 0.4 mL/kg/min, *p* = 0.04) after a median exposure of 150 days [64]. However, this represents low-quality evidence from an uncontrolled case series. The use of PAH-targeted therapies and pulmonary vasodilators for exercise PH is not currently recommended by any guidelines.

Recently, our group provided hypothesis-generating evidence that riociguat may be more effective than sildenafil in improving exercise capacity (6 min walk distance testing) in EIPH in a single-center retrospective study. However, current data remain insufficient to make any definitive conclusions and recommendations [65].

Levosimendan, a calcium sensitizer, shows promise for improving exercise hemodynamics and exercise tolerance in patients with exercise-induced pulmonary hypertension, particularly in the context of PH-HFpEF. The most robust evidence comes from the randomized, placebo-controlled HELP trial, which demonstrated significant hemodynamic improvements and functional benefits [66].

The LEVEL trial is an ongoing phase 3, multicenter, randomized, double-blind, placebo-controlled study evaluating oral levosimendan in patients with PH-HFpEF [67]. This represents the first phase 3 trial investigating whether oral levosimendan can improve exercise capacity in patients with EIPH due to HFpEF.

## 14. Conclusions

Invasive exercise stress testing of the pulmonary circulation is increasingly recognized as a valuable tool for identifying latent or early-stage pulmonary hypertension (PH). Many patients with early pulmonary vascular disease have normal resting hemodynamics, with abnormalities becoming evident only during exercise when cardiac output rises. By assessing pressure–flow relationships, particularly the mPAP–cardiac output slope, exercise testing enables detection of impaired pulmonary vascular reserve and may facilitate earlier diagnosis and risk stratification before overt resting PH develops.

EIPH is currently defined by a mean pulmonary artery pressure (mPAP)/cardiac output (CO) slope > 3 mmHg/L/min and is associated with impaired prognosis, decreased exercise capacity, and may precede the development of manifest PH.

EIPH has been described in association with a range of conditions, including left heart disease, systemic sclerosis, bone morphogenetic protein receptor type 2 (BMPR2) gene mutations, chronic lung disease, and chronic thromboembolic disease. In these settings, EIPH may reflect early or subclinical pulmonary vascular or cardiopulmonary dysfunction, often preceding the development of overt resting pulmonary hypertension.

EIPH may result from pulmonary vasoconstriction, pulmonary vascular remodeling, or increased upstream transmission of pulmonary venous pressure, reflecting either pre-capillary, post-capillary, or combined pathophysiological mechanisms.

EIPH is associated with reduced exercise capacity, may precede the development of overt pulmonary hypertension in a subset of patients, and is linked to reduced survival, underscoring its clinical and prognostic significance.

Further studies are needed to better define the prognostic significance of EIPH and to determine whether early identification and targeted interventions can improve clinical outcomes.

## Figures and Tables

**Table 1:** Exercise hemodynamic parameters used to characterize EIPH phenotype. mPAP = mean pulmonary artery pressure; CO = cardiac output; PAWP = pulmonary artery wedge pressure; EIPH = Exercise induced PH; PH = pulmonary hypertension; HFpEF = heart failure with preserved ejection fraction.

mPAP/CO slope	PAWP/CO slope	Hemodynamic Pattern	Clinical Interpretation
≤3	≤2	Normal	Normal exercise hemodynamics
>3	≤2	Pre-capillary	EIPH due to pulmonary vascular dysfunction
>3	>2	Post-capillary	EIPH due to left-heart disease (i.e., HFpEF)
≤3	>2	Abnormal left-heart response	Isolated abnormal left heart filling pressure response: possible early HFpEF without EIPH

## Data Availability

No new data were created or analyzed in this study. Data sharing is not applicable to this article.
